# A Hazard Analysis of Class I Recalls of Infusion Pumps

**DOI:** 10.2196/10366

**Published:** 2019-05-03

**Authors:** Xuemei Gao, Qiang Wen, Xiaolian Duan, Wei Jin, Xiaohong Tang, Ling Zhong, Shitao Xia, Hailing Feng, Daidi Zhong

**Affiliations:** 1 Bioengineering College Chongqing University Chongqing China; 2 Department of Medical Device Adverse Event Vigilance Chongqing Center for Adverse Drug Reaction Monitoring Chongqing China; 3 Chongqing Academy of Science & Technology Chongqing China

**Keywords:** infusion pump, risk management, equipment failure, hazard analysis and critical control points, man-machine systems

## Abstract

**Background:**

The adverse event report of medical devices is one of the postmarket surveillance tools used by regulators to monitor device performance, detect potential device-related safety issues, and contribute to benefit-risk assessments of these products. However, with the development of the related technologies and market, the number of adverse events has also been on the rise, which in turn results in the need to develop efficient tools that help to analyze adverse events monitoring data and to identify risk signals.

**Objective:**

This study aimed to establish a hazard classification framework of medical devices and to apply it over practical adverse event data on infusion pumps. Subsequently, it aimed to analyze the risks of infusion pumps and to provide a reference for the risk management of this type of device.

**Methods:**

The authors define a general hierarchical classification of medical device hazards. This classification is combined with the Trace Intersecting Theory to form a human-machine-environment interaction model. Such a model was applied to the dataset of 2001 to 2017 class I infusion pump recalls extracted from the Food and Drug Administration (FDA) website. This dataset does not include cases involving illegal factors.

**Results:**

The proposed model was used for conducting hazard analysis on 70 cases of class I infusion pump recalls by the FDA. According to the analytical results, an important source of product technical risk was that the *infusion pumps did not infuse accurate dosage (ie, over- or underdelivery of fluid)*. In addition, energy hazard and product component failure were identified as the major hazard form associated with infusion pump use and as the main direct cause for adverse events in the studied cases, respectively.

**Conclusions:**

The proposed human-machine-environment interaction model, when applied to adverse event data, can help to identify the hazard forms and direct causes of adverse events associated with medical device use.

## Introduction

### Infusion Pumps

Continuous intravenous delivery of drugs with short half-lives, such as inotropic agents and vasodilators, is a recommended technique in acute care [1]. A syringe pump is a device that intravenously infuses fluids, drugs, or nutrients in the patient [2]. The use of infusion pump is helpful as it helps in reducing nurses’ workload and in improving accuracy and efficiency in terms of delivery of drugs or fluids. The purpose of using a syringe pump in clinical settings is to administer an accurate amount of drug or fluid over a relatively long duration, and it can be especially favorable for continuous infusion of very small amounts such as 0.1 mL/hour [3]. In clinical settings, transfusion pumps and syringe pumps are referred to as *infusion pumps*.

The infusion pump system is mainly composed of the following parts: the microcomputer system, the pump component, the detection system, the alarm device, and the input and display devices. The microcomputer system controls and manages the whole system intelligently, prevents the occurrence of incorrect infusion, and sends an alarm signal to the alarm device for sound and light alarms. The pump component is the power source of the liquid injection. The detection system, which is usually made up of different kinds of sensors in different parts, is used to detect the working state of the infusion pump, thereby facilitating the detection of all kinds of abnormalities in time. The alarm device is used to inform the medical and nursing staff of the normal and abnormal states. The input part is used to set the parameters of the infusion, such as the amount of infusion and the speed of the infusion. The display section is responsible for displaying the parameters and the current working state.

The use of infusion pumps was identified as the area with the highest risk, based on incident report data [4]. In unique studies by Keers et al, a higher median medication administration error (MAE, 10 studies used denominators falling within the total opportunity for error [TOE] definition of the 12 studies that examined only intravenous administration) rate was observed for the intravenous route (53% excluding timing errors; interquartile range [IQR]: 27%-58%) without timing errors (n=5) using tolerable negative error (TNE) compared with when all administration routes (56 used the TOE denominator of the 61 studies observing all routes of administration) were studied (20%; IQR: 9%-25%) without timing errors (n=17) using TNE, where each dose could accumulate more than 1 error [5]. Intravenous infusion may present the greatest preventable MAE risk to hospitalized patients [6]. At present, infusion pumps are commonly used in clinics; however, problems exist with respect to the use of infusion pumps in clinics, including discontinued infusion, leakage, inaccuracy of infusion dose, and too fast or too slow infusion speed. According to the clinical needs, analyzing the failure and mode effect of infusion pumps was useful for evaluating the ease of use and ergonomics and evidence-based procurement [7].

The failure modes and infusion errors of infusion pumps are always the top 10 hazards on ECRI Institute’s annual list. Top-ranked hazards of 2017 announced by ECRI Institute focus on infusion errors that can occur when using large-volume infusion pumps [8]. On August 23, 2013, ECRI Institute Patient Safety Organization’s (PSO) clinical engineering staff found certain risks associated with use of infusion pumps during a regular review of device-related events submitted to the PSO. The team saw multiple events at 1 hospital in which an infusion pump had stopped working with no apparent cause. Investigation revealed that a disconnection between the pump module and the personal computer (PC) unit had caused unexpected cessation of infusion therapy for several patients. The problem resulted from corroded or damaged interunit interface connectors [9]. In the ECRI Institute’s PSO Monthly Brief published in February 2015, the patient safety analyst of ECRI Institute PSO, Stephanie Uses, emphasized the potential risk on each phase of the medication use process. She said that there is a risk of confusion among look-alike or sound-alike injection drugs formations, concentrations, and dosages when prescribing the proper one for the patient during the prescribing stage. Risks during the monitoring phase include inadequate monitoring—when patients’ response to insulin is not observed to see if an adjustment in dose is necessary [10].

Thus, effectively decreasing the risks of infusion pumps in clinical settings will be critical for improving the success rate for emergency treatment of patients. In 2010, Zhang et al introduced a generic insulin pump model and a preliminary hazard analysis based on this model [11]; they divided the hazardous situations into 5 categories associated with the generic insulin infusion pump, including therapeutic, energetic, chemical or biological, mechanical, and environmental. Curzon et al established a tool focused on understanding how the design of interactive medical devices (such as infusion pumps, monitors, and diagnostic devices help save lives) can support safety [12]. Masci presented a hazard analysis that identified a substantial set of root causes of use hazards in software design, which is general in the sense that the problematic functionalities are common in broad classes of infusion pumps [13]. Masci et al established a model-based risk analysis methodology that helps manufacturers identify and mitigate use hazards in their products at early stages of the development life cycle [14]. They also presented a generic user interface (UI) architecture, Generic Infusion Pump User Interface, to facilitate the identification and reasoning of use hazards in infusion pumps [14].

### Medical Device Adverse Events

The medical devices, because of their natural characteristics, may bring safety risks, together with health benefits, to the users. The adverse events associated with the qualified postmarket medical devices cause a variety of harms (or potential harms) to the human body under normal operation. These adverse events (including any symptoms, signs, diseases, or the events could result in significant injury or death) do not necessarily have a direct causal relationship with medical devices and can only be temporarily associated with medical devices. The monitoring of medical device adverse events can be useful in warning health care institutions and regulatory bodies on how to use medical devices safely and effectively. All national regulators have established a corresponding data reporting system to actively collect medical device adverse events.

To reduce or avoid the possible risks and damage to human health caused by medical devices, recalling the postmarket defective medical devices is an internationally accepted method for safety management of postmarket medical devices. Being one of the active practitioners of medical device recall, the US Food and Drug Administration (FDA) categorizes all recalls into 3 classes according to the level of hazards caused by medical devices. The class I is defined as dangerous or defective products that predictably could cause serious health problems or death [15]. The recalls are available in the Medical Devices/Safety/List of Recalls on the FDA's official website [16].

In 1972, Professor Elwyn Edwards first proposed the principle of *human* as the center of a particular system interface in security work, including elements such as software, hardware, environment, and liveware (SHEL). The acronym SHEL stands for these 4 elements, and these factors constitute the SHEL model. The human error should be analyzed because of the mismatch between interfacial elements. With respect to the use of medical device risk analysis, in 2011, Long et al established a *medical personnel* –centric medical device risk analysis model based on the SHEL model, called device, environment, liveware, patient, software [17]. Masci et al presented a hazard analysis method that extends Leveson’s System Theoretic Process Analysis with a comprehensive set of causal factor categories so as to provide developers with clear guidelines for systematic identification of use-related hazards associated with medical devices, their causes embedded in UI software design, and safety requirements for mitigating such hazards [18]. Harrison et al concerned with how to demonstrate that a UI software design is compliant with use-related safety requirements, and they established a methodology that aims to demonstrate how to achieve the FDA’s agenda of using formal methods to support the approval process for medical devices [19]. Masci et al established a technique that integrates human cognitive process models and general interaction design principles and uses a model-based approach for systematic exploration of potential hazards [20].

However, from the perspective of medical device supervision, the goal of postmarket medical device risk management is to further discover the causes of unacceptable risks associated with medical devices products through production and postproduction safety data (including medical device adverse events), such as product design, production process, specifications and other issues, and then take appropriate risk control measures, that is, considering *product* as the center of the risk analysis, carrying out evaluation and control process, and making sure that its starting point and foothold are *products*.

Therefore, based on the above research results, this paper presents a hazard classification framework of the medical devices and human-machine-environment interaction model, which was used to analyze 70 cases of FDA class I infusion pump recalls, to identify the direct cause of all risks, then put forward some advice for the life cycle management of infusion pumps.

## Methods

### Overview

Adverse event reports are the main source of data for this study. Our aim here was to find key hazard risk factors and direct causes through the analysis of adverse event reports. Analyzing the hidden risk of medical device based on adverse event report is generally considered as a complicated job. The risk factors cannot be directly extracted if we do not have an appropriate tool to structuralize the content in those reports. For example, in an infusion pump, the application environment is a complex system of human-machine-environment interaction. It is almost impossible for us to identify the hidden risk factors without thoroughly understanding this complex system. Therefore, in this study, we developed a tool that allows the modeling of such a complex system, and then, we used this tool to analyze the hazard of infusion pumps.

This tool was developed based on the Trace Intersecting Theory, which is a widely used generic tool for the analysis of a complex system. However, it is too general to be directly applied to our target—infusion pumps. To better adapt to the characteristics of medical device products, we extended this theory with 5 new types, so that the model could be applied to the risk analysis of medical devices, and then applied it to analyze the infusion pump recalls.

### The Hazard Classification Framework

In terms of the evolution process of safety theory, the early theories of *accident proneness* emphasized the influence of people’s personality characteristics on accidents. Later in 1931, Heinrich put forward the *accident causation theory*, emphasizing that accidents are the result of the interaction of various factors. In 1961 and 1966, Gibson and Haddon introduced a new concept: accidents are incorrect or undesirable energy transfers or releases. At this time, it was discovered that injury accidents could be prevented by controlling energy. In 1969, Surry suggested that people’s mishandling of information might lead to accidents. After the inheritance and development of these ideas by many people, it was found that the unsafe behavior of people or the unsafe state of things is the direct cause of industrial accidents.

**Figure 1:**
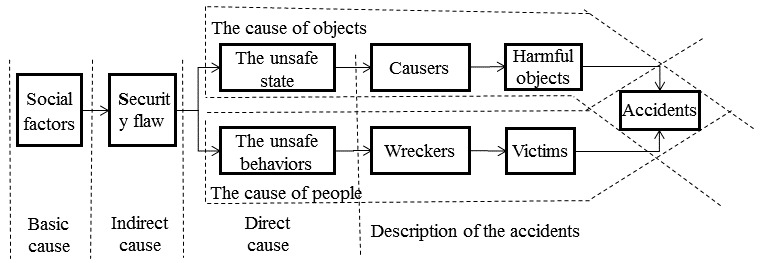
The schematic diagram of the Trace Intersecting Theory.

The Trace Intersecting Theory focuses on the cause of the accident. Such causes can be summarized as equipment’s faults (or defects) and human errors. The intersection of the 2 event chains indicates an accident. The basic idea is that injury accidents are the result of the development of 2 series of interrelated people and things (including the environment). As a result of a variety of factors, the unsafe behaviors of people and the unsafe state of the objects will keep on evolving in their respective trajectories, and accidents will happen at a later point when they meet or interact at a certain time and space (see Figure 1).

**Figure 2:**
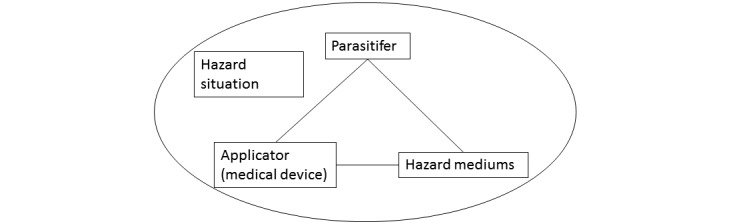
The mechanism of medical device adverse events.

The occurrence mechanism of medical device adverse events consists of 4 types of interactive factors (see Figure 2). Among them, the *parasitifer* is an individual who may be injured, including the patient and/or medical personnel. The *applicator* is the medical device that generates force and transmits or prevents energy, and a *human-machine* relationship is formed between the *applicator* and the *parasitifer*. For the purpose of diagnosis and treatment, the exchange or transmission of material, energy, and information between the human body system and the medical device system will continue. When the material, energy, and information involved in the exchange or transmission exceed the limit tolerance of the human body, a certain type of harm will occur, which we refer to as *hazard mediums*. The *hazard situation* focuses on the conditions or environment in which the injury occurs, that is, the condition and degree of the human body in various hazardous environments.

Modern physics considers that material and energy are the elements of the objective world, but a closer look will find that information is another attribute of the objective world, in addition to the material and energy [21]. Therefore, we use the material, energy, and information as the 3 fundamental elements to model the objective world for the purpose of hazard analysis, but it is difficult to separate material from energy because energy exists in any type of material, and energy cannot live alone without the material being its host. Thus, in the following analysis, the material and energy are merged together and is analyzed as *energy*.

Therefore, medical device adverse events can be divided into 3 types based on different hazard mediums: (1) Type I: Energy hazard; (2) Type II: Information hazard; and (3) Type III: Energy and Information hazard (see Table 1).

The Energy hazard medium is called type I medical device adverse event. It refers to the event wherein medical devices may directly cause human injury in the form of energy under the application environment [22]. The Energy hazard can be further divided into 2 subtypes: the excess energy and the insufficient energy. Among them, the excess energy refers to the scenario when certain kind of energy exceeds the threshold that the humans can bear, which may directly or indirectly lead to the damage of human body. The form of such excess energy can be mechanical energy (Ia-01), radiant energy (Ia-02), thermal energy (Ia-03), electricity (Ia-04), biological and chemical energy (Ia-05), and the others (Ia-06). The insufficient energy refers to an event that may cause human injury directly because of interference in the normal life energy and material exchange, between the human body and the surrounding environment. These cases are in the form of hypoxia, hypothermia, and hydropenia, which can cause exchange impairment between the human body and the surrounding environment (Ib-01), or the failure of life support or first-aid in critically ill patients (Ib-02), and the others (Ib-03).

The Information hazard is called a type II medical device adverse event. It refers to events that may directly cause human injury in the form of information under the application environment. This type of hazard can be further divided into 3 types: incorrect information, insufficient information, and overloaded information, which are in the form of data, text, sound, and image.

The Energy and Information hazard has the characteristics of both the type I and type II hazards at the same time and is called type III medical device adverse event. According to the weight of each constitutional hazard, the type III hazard can be divided into 3 subtypes: the energy-dominant, information-dominant, and dual-culprit. The dual-culprit subtype means that both Energy and Information contribute significantly to the hazard.

**Table 1:** The hazard classification framework of the medical devices.

Hazard classification	Subtype
Type I: Energy hazard	Subtype Ia: (excess energy)
	Subtype Ib: (insufficient energy)
Type II: Information hazard	Subtype IIa: (incorrect information)
	Subtype IIb: (insufficient information)
	Subtype IIc: (overloaded information)
Type III: Energy and Information hazard	Subtype IIIa: (energy-dominant)
	Subtype IIIb: (information-dominant)
	Subtype IIIc: (dual-culprit)

### The Direct Causes Classification

**Figure 3:**
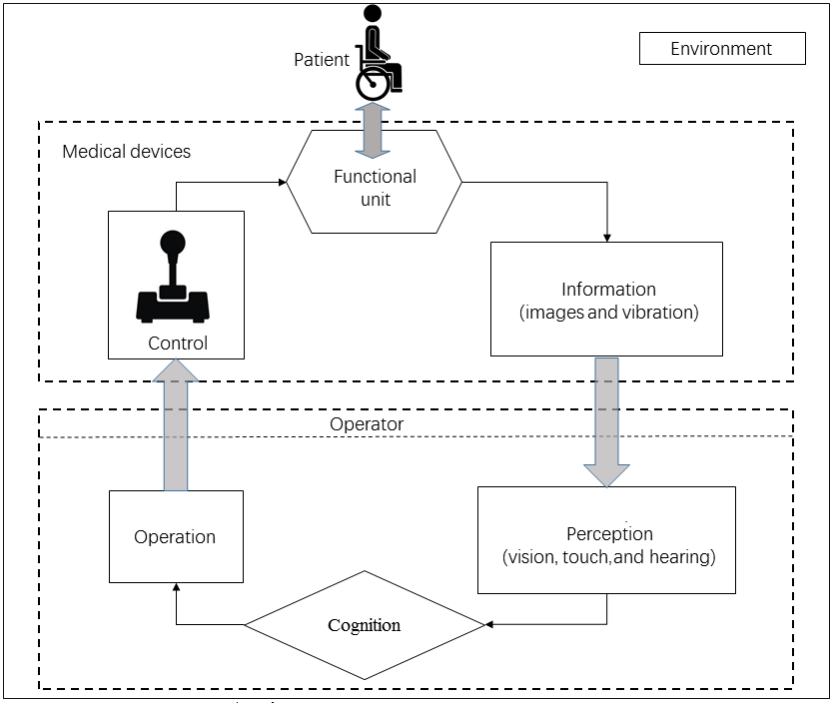
The schematic diagram of the human-machine-environment interaction model.

From the viewpoint of system security, the risk factors of *human-machine-environment* system come from 3 interrelated aspects: *human*, *machine*, and *environment*. In a specific environment, the user has acquired recognition, perception of different information of medical devices, and repeated the actual operation. Through this process, medical devices can be controlled and used to diagnose and treat patients. To describe how a hazard was caused by such interaction among human, medical device, and environment, the authors define a human-machine-environment interaction model (see Figure 3) that contains 5 kinds of direct causes (operator-device, O-D; patient-device, P-D; environment-device, E-D; device, D; and unknown, U). Each direct cause (see Table 2) represents a set of direct causes of certain group of adverse events.

**Table 2:** The type of direct causes.

Direct cause	Description	Main forms
Operator-device	A safety accident that may be caused by a problem in the interaction between the operator and the device	Usability problems: display interface; control interface; HMI^a^ matching (space, seat, and workspace); label or specification; other
Patient-device (P-D)	A safety accident that may be caused by a problem in the interaction between the patient and the device	P-D-1 usability problems: display interface; control interface; HMI matching (space, seat, and workspace); label or specification; other
		P-D-2 internal risk: biocompatibility (blood, tissue, and immunoreaction); tissue or organ infection; tissue or organ damage; other
Environment-device	A safety accident that may be caused by a problem in the interaction between the environment and the device	Effect on the environment or disturbance by other devices: pollution (eg, air pollution); disturbance (eg, electromagnetic interference; other
Device (D)	A safety accident that may be caused by component failure of the device	D-1 (hardware failure); D-2 (software failure)
Unknown	Unknown causes or unexpected injuries	Unknown scientific principle involves multiple chaotic factors and unexpected events

^a^HMI: human-machine interface.

The O-D type direct cause refers to the safety events caused by the problem in interaction between the operator and the device, which is mainly expressed as the availability problems, including display interface, control interface, and label or specification. The P-D type direct cause refers to the safety events caused by the problem in the interaction between the patient and the device, which is mainly expressed as the availability problems and the internal risk. The interpretation of the availability problems is the same as above. The internal risks include biocompatibility (blood, tissue, and immune response), tissue or organ infection, and tissue or organ injury. The E-D type direct cause refers to the safety events caused by the interaction between the environment and the device, which is mainly expressed as the equipment affecting the work environment or being affected by other facilities. The D type direct cause refers to the safety events caused by the failure of the device component, which is mainly expressed as hardware failure and/or software failure. The U type direct cause refers to the safety events caused by unknown causes or unexpected injuries. Among them, O refers to operator, P refers to patient, D refers to device, E refers to environment, and U refers to unknown.

To help readers to better understand the use of the hazard classification framework established in this paper, the following example provides detailed instructions, shown in Multimedia Appendix 1: ID 17. Manufacturer reason for recall: package labeled as an insulin syringe for use with U-100 insulin contains an insulin syringe for use with U-40 insulin. This entails the risk of overdose of insulin. The incident involved 2 aspects of the hazard, including overdose of insulin (Ia-05) and mislabeling (IIa), which is caused by the problem in interaction between the operator and the device (the O-D type direct cause).

**Figure 4:**
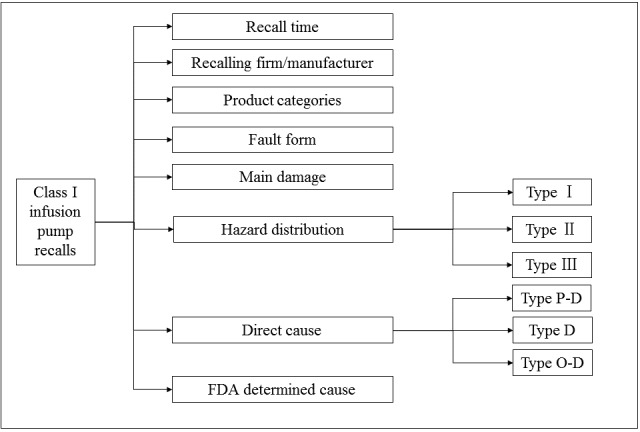
The inferencing pathway of statistical analysis. FDA: Food and Drug Administration. O-D: Operator and device. P-D: Patient and device. D: Failure of the device.

Figure 3 illustrates the pathway of performing statistical analysis over infusion pump recall by leveraging the above human-machine-environment interaction model and the hazard classification framework (see Figure 4).

## Results

### The Basic Information Statistics

**Figure 5:**
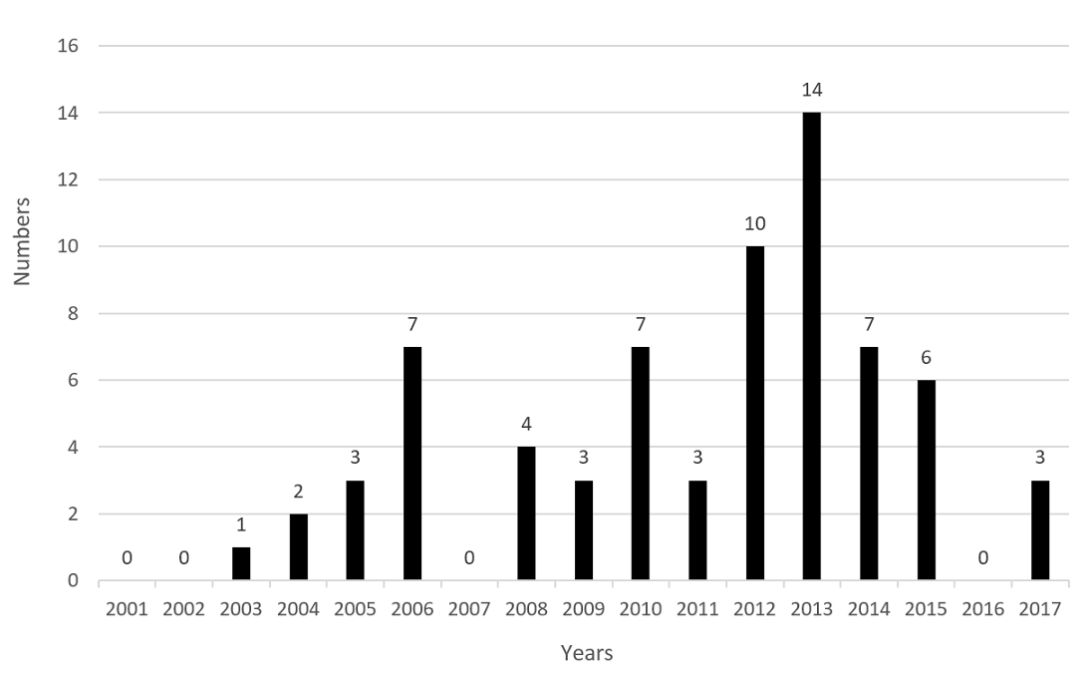
The distribution of recall time.

Figure 5 shows the number of class I infusion pump recalls released by FDA from 2001 to 2017. The largest number of recalls occurred in 2013, which accounted for 20% of the total. The number of recalls from 2001 to 2006 showed a rising trend, and thereafter, a downward trend was observed for 5 years after 2006. The total number of recalls from 2012 to 2015 accounted for 53% of the total, and there was a gradual decline trend after 2013.

Product recalls were mainly issued by the following firms or manufacturers: Medtronic Inc, Hospira Inc, Baxter Healthcare Corp, and CareFusion 303, Inc (see Table 3). The total number of recalls for the 4 companies accounted for 57%. However, the largest number of recalls of a company’s products does not indicate that the company’s products are more risky, because a bigger market share is likely to increase the number of recalls.

Infusion pumps can be divided into the following subtypes: injection pump, elastic pump, and peristaltic pump [23,24]. The most common type of injection pump is the insulin pump. The nutrition pump is an example of the peristaltic pump, and the disposable infusion pump is an example of an elastic pump [23]. Infusion pumps are also categorized into epidural pumps and intravenous pumps. The epidural pump is a topical medication, and the intravenous pump is a systemic medication; the epidural pump can achieve a good analgesic effect with very few drugs, but the catheter is easy to fall off when the patient moves.

Of the 70 cases, 12 (17%) are passive devices, including 6 cases of disposable medical equipment and 6 cases of infusion pump components. The remaining 58 (83%) are active equipment. Infusion pumps make up the maximum proportion, followed by insulin infusion pumps. There were 7 cases of recalls related to infusion pump applications (see Table 4).

There were 17 cases of adverse events caused by *software failures* (see Table 5). The main outcome of equipment faults was product component failure, characterized by sensor failure, pump door breakdown, flow restrictor failure, keypad failure, infusion tube bending or occlusion, the detachment of catheter access port from the main body of the pump, etc. As shown in Table 5, there were many occurrences of power failures and alarm failures (no alarm and false alarm). Furthermore, there can be other problems such as mislabeling, backflow or free flow, and unintended higher flow rate.

Of the 70 cases, 66 described the main damage to patients (see Table 6), which manifested as infection, overdose, underdose, and incorrect treatment. It is known that underdose can result in delay or interruption of infusion therapy, serious injury, or death. Moreover, a drug overdose can lead to serious adverse clinical consequences such as respiratory depression, coma, or death.

**Table 3:** The distribution of recalling firm or manufacturer.

Recalling firm/manufacturer	Recalls, n
Medtronic Inc	14
Hospira Inc	11
Baxter Healthcare Corp	8
CareFusion 303, Inc	7
Disetronic Medical Systems, Inc	3
Animas Corporation	2
B. Braun Medical, Inc	2
Cardinal Health	2
Covidien	2
Insulet Corporation	2
Sigma International General Medical Apparatus, LLC	2
Codman & Shurtleff, Inc.	1
Elite Biomedical Solutions LLC	1
First Medical Source LLC	1
ICU Medical, Inc	1
I-Flow Corporation	1
Iradimed Corporation	1
Manufacturer Codman & Shurtleff, Inc	1
Micromedics, Inc	1
MOOG Medical Devices Group	1
Nurse Assist, Inc	1
Roche Insulin Delivery Systems Inc	1
Smiths Medical ASD, Inc	1
Symbios Medical Products, LLC	1
Tandem Diabetes Care Inc	1
Walkmed Infusion LLC	1

**Table 4:** The list of product categories (N=70).

Product categories	n (%)
Intravenous injection transfusion system	4 (6)
Infusion pump applications	7 (10)
Insulin infusion pump	12 (17)
Infusion pump	47 (67)

**Table 5:** The list of the fault form. A case of a recall may have multiple equipment failures.

Equipment faults	Count, n
Electrical shorting	1
Failure to detect air-in-line conditions	1
Weak seals of the sterile pouches	1
Cartridges leaking	1
Mislabeled	2
Unexpected shutdown	2
Higher flow rate	3
Backflow or free flow	3
Power failure	9
Alarm failures	15
Software failures	17
Component failure	22

**Table 6:** The list of the main damage (N=66).

Hazard	n (%)
Infection	3 (5)
Incorrect treatment	9 (14)
Overdose	18 (27)
Underdose	36 (55)

### The Hazard Classification Statistics

Next, we examined the effect of the hazard classification framework. These data suggest that Energy hazard was the major form of expression (see Table 7).

Certain cases of subtype I hazard may correspond to multiple harmful mediums form, which could be recognized as both the excess energy and insufficient energy. Due to this, the 47 cases of type I hazard in Table 7 actually contain 27 cases of excess energy and 32 cases of insufficient energy (Table 8 shows the corresponding detailed distributions).

The results show that the subtype II hazard (Information hazard) includes 1 case of incorrect information and 3 cases of insufficient information. Moreover, 19 cases of subtype III hazard (Energy and Information hazard) include 14 cases of energy-dominant and 5 cases of information-dominant.

**Table 7:** The distribution list of hazard distribution (N=70).

Hazard classification	n (%)
Ⅰ	47 (67)
Ⅱ	4 (6)
Ⅲ	19 (27)

**Table 8:** The distribution list of type I. A case of a recall may have multiple hazard classifications.

Subtype and energy medium		n
**Ia (n=27)**		
	Ⅰa-01	1
	Ⅰa-03	1
	Ⅰa-05	25
**Ib (n=32)**		
	Ⅰb-02	25
	Ⅰb-03	7

### The Direct Causes Classification Statistics

Finally, we carried out a statistical analysis on the direct cause. There were 72 cases by reason of a case of a recall that may have multiple direct causes. As shown in Table 9, the D type direct cause makes the maximum proportion.

The availability issues can be observed from the O-D type direct cause, including 2 cases of mislabeled and 4 cases of control interface problems. The D type direct cause includes 17 cases of software failures and 45 cases of hardware failures (see Table 10).

We have noticed that the FDA website published the FDA-determined cause. The statistical analysis revealed device design to be the main cause (see Table 11).

**Table 9:** The distribution list of direct cause (N=72).

Direct cause	Statistics, n (%)
Patient-device	2 (3)
Operator-device	6 (8)
Device	64 (89)

**Table 10:** The distribution list of the D type direct cause (N=65).

The D type direct cause	n (%)	Event manifestations
Software failures	17 (26)	Unexpected shutdown, communications errors
Hardware failures	45 (69)	Component failure, material fracture
Invalid information	3 (5)	—^a^

^a^It was difficult to judge if the main form of the case is hardware failure or software failure, but it was certain that it was caused by the component failure of the device.

**Table 11:** The distribution list of Food and Drug Administration–determined cause.

Food and Drug Administration–determined cause	n
Equipment maintenance	1
Labeling design	1
Mix-up of materials or components	1
Packaging process control	1
Pending	1
Process change control	1
Software manufacturing or software deployment	1
Use error	1
Component change control	2
Under investigation by firm	2
Component design or selection	3
Process control	3
Process design	4
Nonconforming material or component	6
Software design	6
Other	7
Device design	28

## Discussion

Overall, our study established a hazard classification framework for medical devices. Through the statistical analysis on the above 70 cases of FDA class I infusion pump recalls, our results confirmed that the key contributor to the product technical risk is that the *infusion pump did not infuse accurate dosage (over- or underdelivery of fluid).*

### Product Component Failures

Most product component failures are caused by device design. The most popular cases within this type of failure are listed below:

1. The *sensor failure* may generate a false alarm or an undetected fluid buildup within the distal line, resulting in delay or interruption of therapy or overinfusion of fluid.
2. The *full or partial occlusion of the infusion tube* may prevent fluid from reaching the patient, causing an interruption of delivery.
3. The normal closure of the *pump door* is closely related to the dosage delivered, which helps the patient to ensure proper treatment process. If the door assembly breaks, it may prevent the door from closing properly; thus, unrestricted flow may occur. If the door cannot be closed, the pump cannot be used, and this will lead to a delay in therapy.
4. The displacement of the *Flow restrictor bead* may be the root cause of the fast flow of contents.
5. The *Luer tube may break at the connection to the pump*, and if this is not noticed by the patient, the patient may receive an underdelivery of drug. A delay or interruption in therapy has the potential to result in a worst-case scenario such as significant injury or death. Similarly, depending on the drug and the dosage delivered, overinfusion also has the potential to result in significant injury or death.
6. In addition, one fact that may explain these defects is that some companies start selling these pumps when they are still in research and development. This was typically the case for Hospira with the Symbiq pump.

### Software Failure

Of the 70 cases of adverse events (Table 10), 17 were caused by *software failures*. Such failures are usually characterized by the following adverse event contents: *wrong instruction*, *error codes*, or *communication errors*. The operator may execute the wrong operation according to the wrong instruction, resulting in an overdose or underdose.

### Alarm Failures

Of the 70 cases of adverse events, 15 were caused by alarm failures, including 5 cases of *false alarm* and 10 cases of *no alarm*. The main forms include (1) pump shutting off during use without warning and (2) *a false visual or audible alarms* causing the infusion pump to stop supplying the fluids to the patient. The fault alarm system may be due to the failure of *internal detector, inability to trigger the alarm*, *the fault of software*, or *lack of regular maintenance*. Alarm hazards are among the top 5 hazards on ECRI Institute’s 2011 list [25]. These studies could help hospitals to enhance their management system, for example, to improve the existing nurse training system, thus to better educate nurses about their shared responsibilities. At the same time, these studies also provide a new strategy to ensure the safe use of medical devices. Nurses should not only pay attention to the operation procedures but also focus on maintenance. In fact, the shortage of nurses is another possible reason for the failure to maintain medical devices. More importantly, manufacturers can also strengthen postmarket maintenance.

### Power Failure

Power failure can result in the situation where the device ceases operation without warning and also loses the data. An incorrect voltage could potentially lead to a loss of communication between the PC unit main processor and the keyboard processor, which can lead to unexpected loss of therapy. Excessive battery discharge can damage the batteries and may further interrupt the therapy. Therefore, we recommend manufacturers to consider designing other backup power and to simplify the operation of replacing batteries.

Altogether, product component failure is the main direct cause of the infusion pump failure. The Energy hazard, containing the excess energy subtype and insufficient energy subtype, is the major form of the hazard of the infusion pump. Among the excess energy type of hazards, *infection* and *overdose* occur most frequently, but the *interruption of infusion therapy* is the hazard that causes the most serious injuries. A substantial part of the hazard of insufficient energy is the *interruption of therapy*, which is mainly caused by *unexpected shutdown*, *power failure*, or *component failures*.

### Limitations of This Research

The biggest problem is that manufacturers, distributors, medical institutions, and device users fail to actively cooperate with the supervision department. Moreover, many steps should be performed by health care institutions before implementing a pump, which can avoid some of the problems faced by infusion pump users. In particular, many defects are not reported to the FDA or other agencies (eg, Health Canada) but directly to the providers of infusion pumps. As a result, many other types of events are not reported, for example, free flow, valve dysfunction, foam in the product because of the mechanism of the pump, and hemolysis. Therefore, there is a lack of sufficient data to further optimize the model in the research work. In addition, influential factors such as the service life of medical devices does not appear in the report, which in turn increases the difficulty of the research.

### Conclusions

With social progress and development of technology, infusion pumps are widely being used in clinical settings. There is a potential safety risk while alleviating the patient’ s suffering, so it is of great significance to ensure proper and safe use of infusion pumps. This study investigated the direct cause of occurrence of infusion pump risks. This may help to provide reference for the infusion pump risk management as well as effective information for safe use and infusion pump safety in clinical environments. To this end, we propose a new data analysis method that can help reveal a single type of adverse events’ risk characteristics and common problems of medical devices based on the Trace Intersecting Theory. It can be used to guide the specific quality monitoring work for the FDA and national authorities to form a complete regulatory system for postmarket medical devices.

We believe that carrying out risk assessment and analysis work for the postmarket medical devices is of great significance, which can optimize the product risk control solutions and have a positive effect on the development of public health.

## Appendix

Multimedia Appendix 1 The hazard classification of 2001 to 2017 class I infusion pump recalls extracted from the Food and Drug Administration website.

## Abbreviations

D: device
E-D: environment-device
FDA: Food and Drug Administration
IQR: interquartile range
MAE: medication administration error
O-D: operator-device
PC: personal computer
P-D: patient-device
PSO: Patient Safety Organization
SHEL: software, hardware, environment, and liveware
TNE: tolerable negative error
TOE: total opportunity for error
U: unknown
UI: user interface
