# Supplementary material for: A Hazard Analysis of Class I Recalls of Infusion Pumps
Source: JMIR Hum Factors. 2019 May 3;6(2):e10366. doi: 10.2196/10366 (PMC6524450; doi:10.2196/10366)
Supplement: Multimedia Appendix 1 [file humanfactors_v6i2e10366_app1.pdf]

Multimedia Appendix 1

ID	Recall Number	Recalling Firm	FDA Determined Cause	Manufacturer Reason for Recall	hazard classification	Direct causes
1	Z-1350-02	Disetronic Medical Systems, Inc.	Other	Some pumps started programming a bolus that was not initiated by the pump user, and the delivery of an unintended bolus was made unless interrupted by the device user.	I a-05	D-2
2	Z-0910-04	Medtronic MiniMed		Possibility of interrupted insulin flow.	I b-03	D-1
3	Z-1334-04	Medtronic Inc, Neurological & Spinal Division	Other	With the Model 8870 software application card versions AAA 02, BBB 04, BBC 02, and BBD 01, which is used in conjunction with the Model 8840 N'Vision Clinician Programmer, users may have mistakenly entered a periodic bolus interval into the minutes field, rather than the hours field, resulting in drug overdoses. This issue is limited to programming the SynchroMed and SynchroMed EL pumps.	I a-05	D-2
4	Z-1060-05	Baxter Healthcare Corp.	Device Design	A hardware problem can cause internal communications errors which halt therapies, generate alarms and communication failure codes.	I b-02	D-1
5	Z-1022-05	Baxter Healthcare Corp.	Device Design	The pumps may experience inadvertent power off, external communications port failures and electronic pump failure codes 402, 403, 533, 535, 599, 810:04 and 810:11.	I b-02	D-1
6	Z-1543-05	Baxter Healthcare Corp.	Labeling design	Swelling of the sealed lead-acid batteries in the infusion pump can cause internal pump damage, and excessive battery discharge can damage the batteries if the pump is left on battery power for an extended period of time after teh Battery Depleted alarm occurs.	I b-02	D-1
7	Z-0445-06	Baxter Healthcare Corp.	Device Design	Interruption of therapy due to battery undercharging, the generation of air-in-line alarms due to IV administration set tugging, and gearbox wear; and underinfusion due to misalignment of the	I b-02	D-1

				pump head components.		
8	Z-1091-2007	Baxter Healthcare Corp.	Software design	A software anomaly is causing newly upgraded Colleague Triple Channel Infusion Pumps to alarm, display an error code (16:310:867:0002) and stop the infusion. This occurs during user programming with all three channels simultaneously infusing fluids. In reported cases, the pump stopped infusing and both an audible and a visual alarm notification were activated.	Ⅲa: Ⅰb-02+ Ⅱa	D-2
9	Z-1132-06	Disetronic Medical Systems, Inc.	Other	The luer tube may break at the lock-tubing connection to the pump, causing an interruption of insulin delivery, which can and has contributed to hyperglycemia.	Ⅰ b-03	D-1
10	Z-1413-06	Disetronic Medical Systems, Inc.	Other	The battery may turn the pump off without warning due to a design change in the battery.	Ⅲa: Ⅰb-03 + Ⅱb	D-1
11	Z-1484-06	Cardinal Health 303 Inc DBA Alaris Products	Software design	This recall was initiated because of a potential for over infusion with all models of the Alaris¿ SE Pumps (formerly the Signature Edition¿ Infusion Pumps) caused by key bounce.	Ⅰ a-05	D-1
12	Z-0022-2007	Medtronic Neuromodulation	Process design	The Catheter Access Port (CAP) on SynchroMed EL pumps manufactured between March and July 1999 may detach from the main body of the pump, which can interrupt drug flow to the target site.	Ⅰ b-02	D-1
13	Z-1414-06	Medtronic Neurological	Other	Tip dislodgement during implantation-Medtronic is recalling Model 8731 Intrathecal Catheter and Model 8598 Intrathecal Catheter Distal Revision Kit because the platinum-iridium tip may be dislodged by the guide wire during implantation.	Ⅰ a-05	D-1
14	Z-1142-2008	Medtronic Neuromodulation	Device Design	Device/Drug Interaction - The company updated the labeling for the devices to include current patient management and treatment recommendations. The company received reports of inflammatory mass formations at or near the distal tip of intrathecal catheters which infuse opioids,	Ⅰ a-05	P-D-2

				baclofen, or chemotherapy drugs into patients.		
15	Z-0744-2008	Medtronic Neuromodulation	Device Design	Pump motor stall due to gear shaft wear.	Ⅰb-02	D-1
16	Z-0292-2009	Animas Corporation	Nonconforming Material/Component	Unintentional rebooting: Pump products exhibit an intermittent loss of power due intermittent loss of contact between battery cap and battery canister resulting in the device resetting. The failure of the battery cap may result in failure of the device to administer insulin therapy which may result in hyperglycemia.	Ⅰb-03	D-1
17	Z-0284-2009	Covidien LP	Process design	Mislabeled: Package labeled as an insulin syringe for use with U-100 insulin contains an insulin syringe for use with U-40 insulin. Risk of overdose of insulin.	Ⅲa: Ⅰa-05 + Ⅱa	O-D
18	Z-1037-2009	Baxter Healthcare Corp.	Device Design	Baxter has identified failure codes that lead to an interruption of therapy, hazards associated with improper cleaning, and damaged battery messages for the Colleague Infusion Pumps.	Ⅰb-02	D-1+D-2
19	Z-1790-2009	Cardinal Health 303 dba Cardinal Health	Device Design	There is a potential risk for medication not being delivered when IV tubing sets are occluded in the Alaris Pump module. This can occur if IV sets are set up in the infusion pumps but not used for an extended period of time. If users fail to clear the occlusion, then the occluded tubing may cause medication not to be delivered. A delay of medication may result in serious injury and/or death.	Ⅲa: Ⅰa-05/ Ⅰb-02+ Ⅱb	D-1
20	Z-1705-2009	Medtronic MiniMed	Process control	Approximately 2% of the infusion sets in the affected lots may not allow the insulin pump to vent properly. Venting is necessary to equalize the pressure in the syringe compartment with the surrounding atmosphere. If the vent does not work properly, this could potentially result in too much or too little insulin being delivered.	Ⅰb-03/ Ⅰa-05	D-1
21	Z-0001-2011	Baxter Healthcare Corp.	Device Design	The FDA sent a letter to Baxter on April 30, 2010, ordering the company to recall and destroy all models of its Colleague	Ⅰb-02	D-2

				Volumetric Infusion Pumps currently in use in the United States. FDA determined that this action is necessary, as Baxter has failed to adequately correct, within a reasonable timeframe, the deficiencies in the Colleague pumps still in use.		
22	Z-1894-2010	Hospira Inc.	Device Design	Hospira has received numerous customer complaints of the Symbiq infusion pump's failure to detect air-in-line conditions during operation.	Ⅱb	D-3
23	Z-2381-2010	Hospira Inc.	Device Design	If the administrator set is removed prior to the cassette carriage fully opening without closing the slide/roller clamp, unrestricted flow and delivery may result.	Ⅲa: Ⅰa-05+ Ⅱb	D-1
24	Z-0128-2011	Hospira Inc.	Device Design	Hospira has identified motor encoder failures in the Symbiq pumping mechanism that causes the infuser to cease operation during infusion therapy, resulting in delay or interruption of infusion therapy.	Ⅰ b-02	D-2
25	Z-0146-2011	Sigma International General Medical Apparatus, Llc	Device Design	The infusion pumps have the potential to fail causing inaccurate flow conditions during use. These conditions range from back flow to free flow, which could result in over-infusion.	Ⅲa: Ⅰb-02+ Ⅱb	D-3
26	Z-0307-2011	Walkmed Infusion LLC	Device Design	Pump door may be in a near shut position, but unlatched, and the "Door Open" alarm may not sound.	Ⅲa: Ⅰa-05+ Ⅱb	D-1
27	Z-0346-2011	Micromedics, Inc.	Packaging process control	Micromedics, Inc is conducting a product recall on a number of medical device products because of weak seals of the sterile pouches potentially resulting in a non-sterile product which may cause transmission of disease or infection.	Ⅰ a-05	D-1
28	Z-1061-2011	Medtronic, Inc. - Neuromodulation	Other	This letter provides important reminders concerning the potential for a pocket fill during a SynchroMed¿ II or SynchroMed EL implantable drug pump refill procedure, and important patient management recommendations that will be added to our product labeling. A pocket fill is the inadvertent injection of all or	Ⅱb	O-D

				some of the prescribed drug into the patients subcutaneous tissue.		
29	Z-3043-2011	Medtronic, Inc. - Neuromodulation	Component design/selection	Medtronic is updating information regarding the potential for reduced battery performance that can lead to sudden loss of therapy in a small percentage of Medtronic Model 8647 SynchroMed II pumps that was communicated with Healthcare providers in July 2009. The purpose of the current communication is to provide updated information regarding the scope and occurrence of this issue.	Ⅰb-02	D-1
30	Z-1483-2011	Roche Insulin Delivery Systems Inc.	Device Design	There is the potential to kink or bend the cannula when inserting the ACCU-CHEK FlexLink Plus infusion set. This can lead to under delivery and elevation of blood glucose levels.	Ⅰb-03/ Ⅰa-05	D-1
31	Z-1667-2012	MOOG Medical Devices Group	Process design	Moog Medical Device Group became aware on March 23, 2012 via customer complaint, of a reversed pump segment on an Administration Set. This malfunction could reverse the flow of fluid or medication backwards from what was intended.	Ⅰb-02	D-1
32	Z-1490-2012	Sigma	Nonconforming Material/Component	Recall expansion; the infusion pumps have the potential to fail causing inaccurate flow conditions during use. These conditions range from back flow to free flow, which could result in over-infusion. Sigma's evaluation of subsequent complaints involving failed bearings indicated that the loss or degradation of bearing lubricant to be a more significant contributor to bearing failure than initiall.	Ⅲa: Ⅰb-02+ Ⅱb	D-1
33	Z-2076-2012	CareFusion 303, Inc.	Component design/selection	The recall was initiated because the Alaris PC unit model 8015 has a component on the PC unit power supply board that is causing an error code(120.4630) "System Error" or "Missing Battery Error" at start up. The error code is accompanied by both an audible alarm and visual error messages on the PC unit screen.	Ⅲb: Ⅰb-02+ Ⅱa	D-2
34	Z-1992-2012	B. Braun Medical,	Device Design	Firm became aware of the potential for breakage of the anti free flow clip catch,	Ⅰa-05	D-1

		Inc.		located on the inside of the pump door, when the IV set anti free flow clip is incorrectly inserted into the pump and the pump door forced closed. If the clip catch is broken and the door opened, free flow protection is still ensured.		
35	Z-2107-2012	Smiths Medical ASD, Inc.	Software design	Smiths Medical has identified a software anomaly in the Medfusion 4000 Syringe Infusion Pump that causes a device history log corruption and triggers a Watchdog Fail-Safe alarm. When the device exhibits this failure mode, visual and audible alarms will sound and the device ceases operation.	Ⅲa: Ⅰb-02+ Ⅱa	D-2
36	Z-1015-2012	B. Braun Medical, Inc.	Device Design	If the IV set anti free flow clip is incorrectly inserted into the pump and the pump door forced closed, the clip catch on the inside of the pump door may break. If the clip catch is broken and the door opened, free flow protection is ensured. If IV set is then removed from pump without closing the roller clamp, the clip may not re-engage the tubing, leaving an open fluid path with free flow pote.	Ⅰb-02	D-1
37	Z-2205-2012	CareFusion 303, Inc.	Device Design	The recall was initiated because Carefusion has identified a potential risk associated with the Alaris Pump module model 8100. The pump module door keypad overlay may separate from the keypad assembly.	Ⅰb-02/ Ⅰa-05	D-1
38	Z-2253-2012	I-Flow Corporation	Device Design	It was determined that in a small quantity of ON-Q pumps with ONDEMAND, the bolus button may not lock in the down position when depressed and/or the orange bolus refill indicator may stay in the lowermost position. As a result, the patient may receive continuous infusion at a rate greater than expected.	Ⅰa-05	D-1
39	Z-0070-2013	Hospira Inc.	Device Design	The Symbiq pump touchscreen may not respond to user selection, may experience a delayed response, or may register a different value from the value selected by the user. Failure of the touchscreen to respond to user input could result in a	Ⅱb	D-1

				delay in therapy or result in over delivery or under delivery of medication if the user does not confirm the programmed values on the pump's confirmation screen prior to starting the infusion.		
40	Z-0161-2013	First Medical Source LLC	Nonconforming Material/Component	Please be aware that this is not a new recall. The firm has taken action; but, due to administrative issues this recall is now being reclassified by the Agency as a Class I. The recall was initiated because First Medical Source has confirmed that these lots may have a higher flow rate than specified. The use of this product may lead to over-administration of drug solutions to the patients.	Ⅰa-05	D-1
41	Z-0350-2014	Hospira Inc.	Device Design	The proximal and distal pressure sensor calibration can drift resulting in the pump failing the Proximal or Distal Occlusion Operational Test, as described in the GemStar Technical Service Manual, or reporting one of the following errors during device setup or infusion: 1) Cassette Check - D; 2) Cassette Check - P; 3) Proximal Occlusion; 4) Distal Occlusion; 5) Pressure Calibration Error; 6) Bad Pressure Sensor Event; 7) Bad Pressure State; 8) Distal Pressure is Out of Range; 9) Proximal Sensor is Out of Range. A pump with this issue may, instead of reporting an error, not detected occlusions or issue false occlusion alarms, which will stop the infusion and invoke visual and audible warnings to the user.	Ⅲb: Ⅰb-02+Ⅰa-05+Ⅱa	D-1+O-D
42	Z-1691-2013	Manufacturer Codman & Shurtleff, Inc.	Use error	Drug flow rates exceeding programmed flow rates.	Ⅰa-05	D-3
43	Z-1874-2013	Iradimed Corporation	Software Manufacturing/Software Deployment	The Dose Error Reduction System (DERS) can indicate an incorrect recommended value for the rate via a specific key sequence during the initial	Ⅰb-02/Ⅰa-05	D-2

				infusion setup. This can result in a risk of over-infusion or under-infusion if the infusion is started with this rate value.		
44	Z-1835-2013	CareFusion 303, Inc.	Process design	CareFusion is recalling the Alaris PC units model 8015 (PC unit), version 9.12, because it is operating at an incorrect voltage and it could potentially experience a loss of communication between the PC Unit main Processor and Keyboard Processor.	I b-02	D-2
45	Z-1691-2013	Codman & Shurtleff, Inc.	Device Design	Miscalibrated Fill Level Sensor may affect dosing level.	III b: I b-02+ II a	D-1
46	Z-1579-2013	Medtronic Neuromodulation	Device Design	Medtronic Neuromodulation is providing Healthcare Providers with safety information and patient management recommendations related to the potential for electrical shorting internal to they SynchroMed infusion pump. An electrical short circuit in a feedthrough may present as a motor stall or low battery reset/alarm and lead to a loss of or reduction in therapy which may result in the return of und.	I b-02	D-1
47	Z-1575-2013	Medtronic Neuromodulation	Device Design	The Sutureless Connector (SC) Intrathecal Catheter connector has been redesigned to reduce the potential for occlusion at the catheter to pump interface. Medtronic is removing the unused products from the market that were manufactured with the previous design, and recommend the previous design no longer be used due to greater potential for misalignment and subsequent occlusion.	I b-02	D-1
48	Z-1570-2013	Medtronic Neuromodulation	Device Design	Medtronic is providing Healthcare Professionals with important safety information and patient management recommendations regarding the unintended delivery of drug during the priming bolus function for the SynchroMed implantable infusion pump. The unintended delivery of drug can contribute to patient overdose or underdose symptoms which may be	I a-05	D-2

				clinically relevant.		
49	Z-1170-2013	Hospira Inc.	Device Design	Pump shutting off during use without warning.	IIIa: Ⅰb-02+ Ⅱb	D-1
50	Z-1159-2013	Hospira Inc.	Device Design	Battery level lower then 2.4 volts results in corrupt history log and loss of data for preview therapy's parameters and causes an 11/004 error resulting in the pump not being able to be used.	Ⅲ b: Ⅰ b-02+ Ⅱ b	D-1
51	Z-1098-2013	Carefusion Corporation	Software design	The recall was initiated because Carefusion has received reports of a communication error on the Alaris PC unit model 8015 with software version 9.12 when the Alaris EtCO2 module or Alaris SpO2 module is attached.	Ⅰ b-02	D-2
52	Z-0940-2013	Symbios Medical Products, LLC	Pending	Received 2 complaints out of 458 distributed pumps in this lot, where flow restrictor bead became displaced, which were reported to permit fast flow of contents.	Ⅰ a-05	D-1
53	Z-1668-2013	Medtronic MiniMed	Device Design	Medtronic is recalling the Medtronic MiniMed Paradigm Medtronic is recalling the Medtronic MiniMed Paradigm Insulin Infusion sets because of a potential safety issue that can occur if insulin or other fluids come in contact with the inside of Medtronic Paradigm infusion sets. Under certain conditions, the infusion set may malfunction and deliver an incorrect volume of insulin.	Ⅰ a-05	D-1
54	Z-0993-2013	Animas Corporation	Nonconforming Material/Component	Animas has identified a component issue affecting a small supply of the Animas(R) 2020 insulin pumps. The component issue may trigger the pumps to sound a false alarm or warning related to one of the following: - Loss of prime; -Occlusion; or -No cartridge detected.	Ⅲ b: Ⅰ a-05+ Ⅱ a	O-D
55	Z-0095-2015	Hospira Inc.	Process control	The GemStar Infusion pump may not receive direct current (DC) power from the power supply. In one instance, smoke was observed and the GemStar pump was operating on battery power while connected to the 3 volt DC power supply.	Ⅰ a-01+ Ⅰ a-03+ Ⅰ a-05+ Ⅰ b-02	D-1
56	Z-2622-	ICU	Nonconfor	ICU is recalling the ConMed Stat 2 flow	Ⅰ a-	D-1

	2014	Medical, Inc.	ming Material/Component	controller because it may deliver a significantly higher flow rate than was intended.	05	
57	Z-1567-2014	CareFusion 303, Inc.	Software design	CareFusion is recalling the Alaris Pump model 8100 version 9.1.18 because it may have a software issue that results in situation where the pump module will not properly delay an infusion when the "Delay Until" option or "Multidose" feature is used.	Ⅰb-02/Ⅰa-05	D-2
58	Z-1512-2014	Hospira Inc.	Device Design	There are two situations that may occur when using the GemStar Docking Station, List Number 13075-XX-XX, in conjunction with the GemStar infusion pump: 1) when the Docking Station is used in conjunction with a GemStar Phase 3 pump (List 13000-XX, 13100-XX, or 13150-XX) the potential exists for the GemStar Phase 3 pump to fail to power up while connected to the Docking Station, and 2) when either a GemStar Phase 3 (List 13000-XX, 13100-XX, or 13150-XX)or GemStar Phase 4 pump (List 13086-XX, 13087-XX, or 13088-XX) is used in conjunction with both a Docking Station and an External Battery Pack accessory (List 13073-XX) there exists a possibility that the GemStar pump will display error code 11/003 and give an audible alarm indicating excessive input voltage from the external sources. If the GemStar pump detects what is perceived to be more than 3.6 Volts measured on the external voltage input, the pump will stop the infusion and alarm both with an audible sound as well as a visual alarm.	Ⅰb-02	D-2
59	Z-1483-2014	Hospira Inc.	Component design/selection	Broken door assemblies on the Hospira/Abbott Acclaim Encore infusion pumps. If the door assembly breaks, it may prevent the door from closing properly and unrestricted flow may occur. If the door cannot be closed, the pump cannot be used which can result in a delay in therapy.	Ⅰb-02/Ⅰa-05	D-1
60	Z-1484-	Baxter	Software	Baxter Healthcare Corporation has issued	Ⅰb-	D-2

	2014	Healthcare Corp.	design	an Urgent Device Correction for the SIGMA SPECTRUM Volumetric Infusion Pump with Master Drug Library due to repeated System Error 322 occurrences.	02	
61	Z-0827-2014	Tandem Diabetes Care Inc	Equipment maintenance	Tandem Diabetes Care¿, Inc. announced that it is initiating a voluntary recall of specific lots of insulin cartridges that are used with the t:slim¿ Insulin Pump. The affected cartridges may be at risk for leaking. The cause of the recall was identified during Tandems internal product testing, and has not been associated with any complaints or adverse events reported by customers.	Ⅰb-03/ Ⅰa-05	D-1
62	Z-0393-2016	Insulet Corporation	Process change control	Pod's needle mechanism fails to deploy or there is a delay in the deployment of the needle mechanism.	Ⅰb-03	D-1
63	Z-1071-2015	Hospira Inc.	Component change control	One lot of alarm assemblies used in Plum A+ and Plum A+3 infusion pumps may fail to sound at all volume levels.	Ⅲa: Ⅰb-02+ Ⅱb	D-1
64	Z-1018-2015	Covidien	Mixed-up of materials/components	A manufacturing error resulted in the risk of incorrect proximal and distal balloon inflation port identification on the units. Units have been identified to have the distal balloon inflation port incorrectly labeled as proximal, and, the proximal balloon port incorrectly labeled as distal.	Ⅱa	O-D
65	Z-2362-2015	CareFusion 303, Inc.	Process control	Channel Error code is displayed on the PC unit with an audio and visual alarm, and on the syringe module. After the error is cleared on the PCU, the syringe pump is unresponsive to key presses until the next power cycle, or the module is detached and reattached.	Ⅲa: Ⅰb-02+ Ⅱa	D-2
66	Z-2371-2015	Elite Biomedical Solutions LLC	Other	Administration of inappropriate quantities of fluid can result, with the potential to cause injury or death.	Ⅰb-02/ Ⅰa-05	D-1

67	Z-2484-2015	Insulet Corporation	Nonconforming Material/Component	OmniPods¿ (Pods) have a higher rate of failure causing: Cannula fails to deploy/retract causing insulin to be pumped or the audible alarm is displayed on the PDM and pod will not deliver insulin.	IIIa: I b-03 + II a+ II b	D-1+O-D
68	Z-0926-2017	Nurse Assist, Inc	Under Investigation by firm	Potential contamination with B. cepacia.	I a-05	P-D-2
69	Z-0950-2017	CareFusion 303, Inc.	Component change control	During an infusion, a false Air-in-Line (AIL) alarm would cause the infusion to be interrupted.	III b: I b-02+ II a	D-1
70	Z-0788-2017	Medtronic Neuromodulation	Under Investigation by firm	Medtronic is following up to a May 2013 communication regarding the Priming Bolus function for the SynchroMed Infusion System. Medtronic is updating the Model 8870 software application card (to version AAU01) and the SynchroMed pump labeling to address the priming bolus issue.	I b-02/ I a-05	D-2
